# Supplementary material for: Prevalence of Vitamin D and Calcium Deficiency and Insufficiency in Women of Childbearing Age and Associated Risk Factors: A Systematic Review and Meta-Analysis
Source: Nutrients. 2022 Oct 17;14(20):4351. doi: 10.3390/nu14204351 (PMC9612098; doi:10.3390/nu14204351)
Supplement: Supplementary file 1 [file nutrients-14-04351-s001.zip › nutrients-1905004-supplementary.pdf]

Table S1. Prevalence and risk factors associated with vitamin D deficiency in pregnant Brazilian women.

Author/Year/Location/ Region.	Population*/Design	Technique/Classification Deficiency Vitamin D (ng/mL/ nmol/L)	Prevalence (%)	Investigated variables	Associated variables
Prado/2015 [36] Viçosa-Minas Gerais<br><br>Southeast	226 women, 88.5% 20-44 years, 11.5% teenagers/cross-sectional	Chemiluminescence/severe deficiency, <10 ng/mL(25 nmol/L); deficiency, <20 ng/mL(50 nmol/L); insufficiency, 20-30 ng/mL (50 nmol/L)	Severe deficiency: 15%; deficiency: 27%; insufficiency: 58%	Women: age, skin color, place of residence, parity, supplementation, US, SE, education level, MS, TD, AP, Ca, PTH, P	No association
Pereira-Santos/2017[33] San Antonio de Jesus-Bahia<br><br>Northeast	190 women, 18-45 years/cross-sectional	Chemiluminescence/deficiency, <50 nmol/L 25(OH)D ; insufficiency, ≥50-<75 nmol/L 25(OH)D	Deficiency, 14.2%; insufficiency, 44.7%	Age, MFI, YS, skin color, MS, GA, number of weekly SEs, region of body exposed to sun, SY, means of transport	Adjusted analysis of VD deficiency: marital status [married/in a relationship]: RP=2.53 (1.01-6.35); body region exposed to sun [face and hands]: RP=2.99 (1.3-6.63); means of transport [vehicles]: RP= 2.79 (1.06-7.31); collection EA [winter]: RP=1.54 (1.01-2.35)
Figueiredo/2017[37] Rio de Janeiro<br><br>Southeast	199 women, 20-40 years/cohort	Liquid chromatography/ESG: deficiency, <50 nmol/L; insufficiency, 50-<75 nmol/L; IOM: deficiency, <30 nmol/L; insufficiency, 30-<50 nmol/L	ESG: deficiency/trimester: 1st = 16.1%; 2nd = 11.2%; 3rd = 10.2%; insufficiency/trimester: 1st = 70.4%; 2nd = 41%; 3rd = 33.9% IOM: deficiency/trimester	Age, skin color, YS, PCI, parity, smoking in the 1st trimester, AI in the 1st trimester, PA before pregnancy, SY, daily calcium and VD intake	Greater longitudinal increases in 25(OH)D concentration up to the third trimester in those with insufficiency in early pregnancy (p<0.006); longitudinal increase in 25(OH)D concentrations until the third trimester in women who started pregnancy in winter (p<0.001) and spring (p<0.001)

			: 1st = 2.0%; 2nd = 0%; 3rd = 0.6%		
Chrisostomo/2018 [34] Curitiba-Paraná<br><br>South	487 women, 18-40 years/cross-sectional	Chemiluminescence/Endocrine Society: deficiency, <49.92 nmol/L; insufficiency, 49.92-72.38 nmol/L	Deficiency and insufficiency, 82.3%	Age, ethnic origin, skin phototype according to the Fitzpatrick classification, tobacco exposure, AI, YS, PCI, SY; clinical data: preeclampsia, DM, HIV, BMI, medication use, stage of pregnancy, parity, number of spontaneous abortions, NP	VD deficiency and insufficiency: preeclampsia (OR: 1.66/95% CI: 1.05-2.63); blood collection in summer associated with higher mean VD (p<0.001)
Souza/2019 [35] São Luís-Maranhão<br><br>Northeast	174 women, 14-40 years/cross-sectional	Immunoassay/insufficiency, ≥20-<30ng/mL; deficiency, <20ng/mL	Deficiency, 23.6%; insufficiency, 53.4%	Marital status, skin color, PCI, religion, sunscreen use, adolescence, NP, gestational trimester	Lower mean VD in evangelical women (p=0.02) and primiparous women (p=0.03); association between HVD and adolescents (p=0.02), primiparous women (p=0.01); inverse relationship between HVD and income (p<0.01)

*Population includes sample size, age group, and sample origin. **Data obtained by contacting authors. Abbreviations: AI: alcohol intake; AP: alkaline phosphatase; BMI: body mass index; DM: diabetes mellitus; ESG: Environmental, social, and corporate governance; GA: gestational age; HC: head circumference; HIV: Human immunodeficiency virus; HVD: hypovitaminosis D; IOM: Institute of Medicine; MFI: monthly family income; MS: marital status; NP: number of pregnancies; PA: practice of physical activity; PCI: per capita income; PTH: parathyroid hormone; RHI: reactive hyperemia index; SE: sun exposure; RP: prevalence ratio; SY: season of the year; TD: type of delivery; US: use of sunscreen; VD: vitamin D; VDD: vitamin D deficiency; YS: years of study.

Table S2. Prevalence and risk factors associated with vitamin D deficiency in non-pregnant Brazilian women of childbearing age.

Author/Year/Location/Region	Population*/design	Technique/classification Deficiency Vitamin D (ng/mL/ nmol/L)	Prevalence (%)	Investigated variables	Associated variables
Peters/2009 [42] Indaiatuba-SP Southeast	99 women, 16-20 years/cross-sectional	Radioimmunoassay/deficiency, ≤10 ng/mL; insufficiency, >10-<30 ng/mL	Deficiency, 0%; insufficiency, 60.6%	Sunscreen, physical exercise, exposure sun, DBS, SBP	No significant association
Santos**/2012 [43] Curitiba-PR, Porto Alegre-RS South	27 women, 15-18 years/cross-sectional	Chemiluminescence/deficiency, <20 ng/mL; insufficiency, 20-29.9 ng/mL	Deficiency, 25.9%; insufficiency, 55.6%	Age, height, BMI, age at menarche, age at thelarche, SY, genotype, WC	No association between season at the time of blood collection and serum VD
Lopes/2015 [47] São Paulo-SP Southeast	97 women, 14-18 years/cross-sectional	Liquid chromatography/deficiency, <20 ng/mL; insufficiency, 20-29.9 ng/mL	Deficiency and insufficiency, 63.9%	Not investigated specifically for females	
Ferreira/2015 [38] Rio de Janeiro-RJ Southeast	73 women, 18-50 years /cross-sectional	Radioimmunoassay/deficiency, <20 ng/mL	Deficiency, 16.43%	Age, skin color, AI, daily Ca consumption, creatinine, TPL, albumin, globulin, intracellular Ca, serum Ca, ionic Ca, urinary Ca/creatinine, PTH, W, BMI, BF, WC, HC, WC/HC ratio, WC/height ratio, glucose, insulin, HOMA-IR, TCL,	Analysis adjusted for age, BMI, and WC; intake of energy, protein, carbohydrates, lipids, and Ca and intracellular Ca serum PTH, calcitriol, and Ca; association with serum glucose (p=0.03) and HOMA-IR (p=0.04)

				HDL, LDL, TG, leptin, adiponectin, hs-CRP, RHI, SBP, DBP.	
Maciel/2017 [48] Jacareí-SP<br><br>Southeast	15 women, 20-40 years, retrospective cross-sectional	No standardization of clinical analysis/deficiency, <20 ng/mL; insufficiency, 20-29.9 ng/mL	Deficiency, 20%; insufficiency, 40%	not investigated	-
Araújo/2017 [39] João Pessoa-PB<br><br>Northeast	135 women, 15-19 years/cross-sectional	Chemiluminescence/deficiency, <20 ng/mL; insufficiency, 20-29.9 ng/mL	Deficiency and insufficiency, 74.1%	Age, skin color, daily hours of sleep, SE, PA, daily VD intake, weight, height, BMI	Ca and VD:p<0.001<br>Ca and HVD: PR=1.24 (95% CI, 1.0-1.53)
Lopes/2017 [44] Brasilia DF<br><br>Midwest	369 women, 21 -47 years, Armed Forces Hospital/cross-sectional	Chemiluminescence/deficiency, <20 ng/mL; insufficiency, 20-30 ng/mL	Deficiency, 32%; insufficiency, 49.1%	Infertility (low ovarian reserve, PCOS, tubal factors, endometriosis, multiple factors, unexplained infertility)	no associations
Santos/2019 [45] Brazil<br><br>Southern	84 perimenopausal women, population-based study of southern Brazil/cross-sectional	Chemiluminescence/ deficiency, <20 ng/mL	Deficiency, 41.67%	BP, weight, height, WC, BMI, TCL, LDL, HDL, TG, HOMA-IR, estradiol, testosterone, SHBG, PTH, VDBP, albumin	no associations
Vivan**/2019 [40] Porto Alegre-RS<br><br>South	180 women, 18-40 years, candidates for bariatric surgery, mean BMI of 49kg/m², Hospital das	Chemiluminescence/ deficiency, <20 ng/mL; insufficiency, 20-29.9 ng/mL	Deficiency, 11,1%; insufficiency, 68.3%	Age, sex, skin color, educational level, SY, BMI, SAH, DM, multiple comorbidities, use of	Association between VDD and non-white skin (p=0.000), diabetes (p=0.033), use of Ca channel blocker (p=0.012), Hb1Ac (p=0.014), FBG

	Clínicas of Porto Alegre/cross-sectional			thiazide diuretics, use of Ca channel blocker, Hb1Ac, FBG	(p=0.003), autumn-winter (p=0.002), BMI (p=0.003)
Fonseca Valle/2019 [41] Rio de Janeiro-RJ<br><br>Southeast	54 women, 12-19 years, Center for Adolescent Health Studies/cross-sectional	HPLC/ deficiency, <20 ng/mL	Deficiency, 31.48%	Age, weight, height, BMI, WC, waist-to-height ratio, CI, PTH, LDL, HDL, TCL, TG, SBP, DBS, FBG, Hb1Ac, SI, HOMA-IR,	Weight, BMI, WC, WHR, SBP, and HOMA-IR were associated with VDD; there was no statistically significant difference for DBP, CI, glucose, insulin, or PTH
Mendes/2020 [49] several cities in Brazil	79 women, 15-49 years/cross-sectional	HPLC-MS/MS/ deficiency, <20 ng/mL; insufficiency, <30 ng/mL	Deficiency, 39.4%; insufficiency, 69.3%	Not investigated	-
Segheto**/2021 [46] Viçosa-Minas Gerais<br><br>Southeast	244 women, 20-59 years (considered fertile)/cross-sectional	Chemiluminescence/deficiency, <20 ng/mL; insufficiency, 20-29.9 ng/mL	Deficiency, 15.57%; insufficiency, 38.52%	Bone mass, total bone mineral content	No significant association

*Population: includes number, age group, and sample origin. **Data obtained by contacting authors. Abbreviations: AI: alcohol intake; BF: body fat (%); BMI: body mass index; BP: blood pressure; CI: conicity index; DBP: diastolic blood pressure; DM: diabetes mellitus; FBG: fasting blood glucose; Hb1Ac: glycated hemoglobin; HC: hip circumference; HDL: high-density lipoprotein; HOMA-IR: homeostatic model assessment for insulin resistance; HPLC: high-performance liquid chromatography; hs-CRP: high sensitivity C-reactive protein; HVD: hypovitaminosis D; LDL: low-density lipoprotein; PA: practice of physical activity; PCOS: polycystic ovary syndrome; PR: prevalence ratio; PTH: parathyroid hormone; RHI: reactive hyperemia index; SAH: systemic arterial hypertension; SBP: systolic blood pressure; SHBG: sex hormone binding globulin; SI: serum insulin; TCL: total cholesterol levels; TG: triglycerides; TPL: total protein levels; UERJ: State University of Rio de Janeiro VD: vitamin D; VDBP: vitamin D binding protein; VDD: vitamin D deficiency; VLDL: Very low density lipoprotein; WC: waist circumference.

Table S3. Prevalence and factors associated with Ca deficiency in Brazilian pregnant or non-pregnant women of childbearing age.

Author/Year/ Location/Region	Population*/design	Technique/classification Ca deficiency (mg/dL)	Prevalence	Investigated variables	Associated variables
Prado/2015 [36] Viçosa-MG Southeast	226 women, 20-44 years: 88.5%, 11.5% teenagers, pregnant women, Hospital of the municipality of Viçosa/cross-sectional	Calcium-Arsenazo III colorimetric endpoint quantification/hypocalcemia, Ca <8.8 mg/dL	Hypocalcemia: 15.5%	No association with hypocalcemia was investigated.	NR
Peters/2009 [42] São Paulo-SP Southeast	99 women, 16-20 years, Public School of Indaiatuba-SP/cross-sectional	Radioimmunoassay/hypocalcemia, Ca <8.6 mg/dL	Mean: 9.4 mg/dL	No association with hypocalcemia was investigated.	NR

*Population includes number, age group, and sample origin. Legend: Ca: Calcium; NR: not reported.
